# Fascaplysin Exerts Anti-Cancer Effects through the Downregulation of Survivin and HIF-1α and Inhibition of VEGFR2 and TRKA

**DOI:** 10.3390/ijms18102074

**Published:** 2017-09-29

**Authors:** Taek-In Oh, Yoon-Mi Lee, Taek-Jin Nam, Young-San Ko, Shinmee Mah, Jinhee Kim, Younghoon Kim, Rallabandi Harikrishna Reddy, Young Jun Kim, Sungwoo Hong, Ji-Hong Lim

**Affiliations:** 1 Department of Biomedical Chemistry, College of Biomedical & Health Science, Konkuk University, Chungju 27478, Chungbuk, Korea; dk1050@kku.ac.kr (T.-I.O.); tj1994s@naver.com (T.-J.N.); harikrishnareddy.6@gmail.com (R.H.R.); ykim@kku.ac.kr (Y.J.K.); 2 Department of Food Bioscience, College of Biomedical & Health Science, Konkuk University, Chungju 27478, Chungbuk, Korea; yoonmilee@kku.ac.kr; 3 Nanotechnology Research Center, Konkuk University, Chungju 27478, Chungbuk, Korea; 4 Department of Anatomy, Seoul National University College of Medicine, Seoul 03080, Korea; rhdudtks@snu.ac.kr; 5 Center for Catalytic Hydrocarbon Functionalizations, Institute for Basic Science (IBS), Daejeon 34141, Korea; msm115@kaist.ac.kr (S.M.); chem88@kaist.ac.kr (J.K.); hongorg@kaist.ac.kr (S.H.); 6 Department of Chemistry, Korea Advanced Institute of Science and Technology (KAIST), Daejeon 34141, Korea; 7 Department of Pathology, Seoul National University College of Medicine, Seoul 03080, Korea; manofme@naver.com

**Keywords:** fascaplysin, survivin, VEGFR2, TRKA, TRAIL

## Abstract

Fascaplysin has been reported to exert anti-cancer effects by inhibiting cyclin-dependent kinase 4 (CDK4); however, the precise mode of action by which fascaplysin suppresses tumor growth is not clear. Here, we found that fascaplysin has stronger anti-cancer effects than other CDK4 inhibitors, including PD0332991 and LY2835219, on lung cancer cells that are wild-type or null for retinoblastoma (RB), indicating that unknown target molecules might be involved in the inhibition of tumor growth by fascaplysin. Fascaplysin treatment significantly decreased tumor angiogenesis and increased cleaved-caspase-3 in xenografted tumor tissues. In addition, survivin and HIF-1α were downregulated in vitro and in vivo by suppressing 4EBP1-p70S6K1 axis-mediated de novo protein synthesis. Kinase screening assays and drug-protein docking simulation studies demonstrated that fascaplysin strongly inhibited vascular endothelial growth factor receptor 2 (VEGFR2) and tropomyosin-related kinase A (TRKA) via DFG-out non-competitive inhibition. Overall, these results suggest that fascaplysin inhibits TRKA and VEGFR2 and downregulates survivin and HIF-1α, resulting in suppression of tumor growth. Fascaplysin, therefore, represents a potential therapeutic approach for the treatment of multiple types of solid cancer.

## 1. Introduction

As a promising strategy for cancer treatment, recent studies have focused on the development of drugs for inhibiting receptor tyrosine kinases (RTKs), such as the epidermal growth factor receptor (EGFR), vascular endothelial growth factor receptors (VEGFRs), platelet-derived growth factor receptor (PDGFR), and the tropomyosin-related kinase (TRK) family [1]. In particular, TRKA, a member of the RTK family, is emerging as a potential therapeutic target for the treatment of human cancers, owing to its oncogenic function associated with cell growth, proliferation, survival, and evasion of apoptosis [2]. Moreover, it has been reported that synthetic azaindole derivatives, such as TRKA inhibitors, confer anti-cancer effects by suppressing v-Akt murine thymoma viral oncogene (AKT) -mediated growth signaling in vitro [3]. Since VEGFR2 is closely linked to pathological angiogenesis, including cancer and inflammation, an efficient inhibitor targeting VEGFR2 would be useful for the treatment of human cancer [4]. Indeed, a recent preclinical study has demonstrated that tubeimoside-1 can suppress cancer growth and angiogenesis by attenuating mTOR-AKT signaling in non-small cell lung cancer [5].

Fascaplysin, originally identified as a natural compound from the marine sponge, and its synthetic derivatives have been reported as potential CDK4 inhibitors [6,7,8]. However, the anti-cancer effects of fascaplysin and its derivatives via CDK4 have not been fully studied. Kumar et al. recently suggested that fascaplysin induces apoptosis and autophagy by suppressing the PI3K-AKT-mTOR signaling cascade in HL-60 cells [9], indicating that the identification of unknown molecules, namely key targets for the development of fascaplysin-derived anti-cancer drugs, is necessary. 

It is becoming clear that survivin, as an anti-apoptotic factor owing to its suppressive function in the pro-apoptotic signal-mediated caspase cascade, is an attractive therapeutic target because of its differential expression in cancer versus normal tissues, which allows for specificity in cancer therapies [10,11]. Indeed, targeting survivin with genetic silencing using antisense oligonucleotides or pharmacological inhibition using small molecules, such as YM155, induced apoptosis by triggering caspase activation in tumor cell lines whereas did not in normal cells [12]. Moreover, many reports suggest that the suppression of survivin sensitizes tumor necrosis factor-related apoptosis-inducing ligand (TRAIL)-based cancer therapy that requires the incorporation of other anti-cancer agents because of its limited anti-cancer efficacy due to TRAIL-resistance [13,14,15,16,17].

Furthermore, 4EBP1 and p70S6K1, which are regulated by growth factor-mediated mTOR signaling, control tumor development and progression through eIF4E-mediated cap-dependent translation of tumor promoting genes, such as survivin, HIF-1α, cyclin D1, and c-Myc [18,19]. Several preclinical studies have reported potential therapeutic tools for the treatment of human cancers, that target the translation machinery, such as mTOR inhibitors (e.g., Rapamycin, Torin 1), eIF4E inhibitors (e.g., 4EGI-1, 4E1RCat), and MNK inhibitors (e.g., Cercosporamide, CGP57380) [20]. 

In the present study, we determined the dependency of the CDK4-retinoblastoma (CDK4-RB) axis in the suppression of cancer growth by fascaplysin, and identified TRKA and VEGFR2 as novel target molecules, which are directly targeted by fascaplysin. Our results showed that fascaplysin downregulates survivin and HIF-1α, resulting in increased apoptosis and decreased angiogenesis due to the suppression of 4EBP1-mediated cap-dependent translation.

## 2. Results

### 2.1. Fascaplysin Rapidly Decreases Cell Viability and Induces Apoptosis Independently of CDK4-RB Inhibition

**Figure 1:**
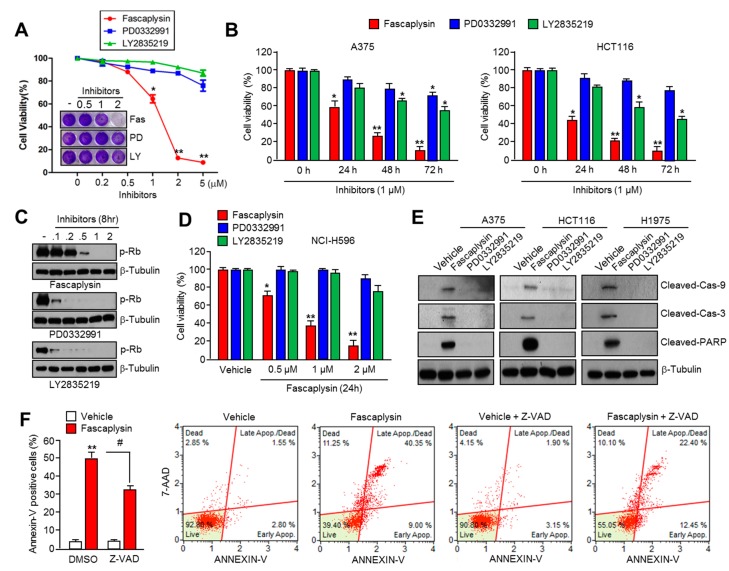
Fascaplysin rapidly induced apoptosis independently of the CDK4- retinoblastoma (CDK4-RB) axis. (**A**) The growth inhibition by CDK4 inhibitors, such as fascaplysin, PD0332991, or LY2835219 in A375 malignant melanoma cells for 24 h. Crystal violet staining images are shown. Values represent the mean ± SD of three independent experiments performed in triplicate; * *p* < 0.05 and ** *p* < 0.01; (**B**) The growth inhibition by fascaplysin in A375 and HCT116 colorectal cancer cells for 24, 48, and 72 h. Values represent mean ± SD of three independent experiments performed in triplicate; * *p* < 0.05 and ** *p* < 0.01; (**C**) A375 cells were treated with various concentrations (0.1–2 µM) of CDK4 inhibitors for 8 h, and then phosphorylated-RB proteins were determined by Western blotting; (**D**) Cell viability in RB-null NCI-H596 in the absence or presence of CDK4 inhibitors. Values represent mean ± SD of three independent experiments performed in triplicate; * *p* < 0.05 and ** *p* < 0.01; (**E**) The cells were treated with 1 µM of CDK4 inhibitors for 24 h, and then cleaved-caspase-9, -3, and Poly (ADP-ribose) polymerase (PARP) were determined by western blotting; (**F**) Retinoblastoma (RB)-null NCI-H596 cells were incubated with 1 µM of fascaplysin for 48 h in the absence or presence of the pan-caspase inhibitor *Z*-VAD-FMK. After annexin-V staining, the population of cells was determined by FACS analysis. Values represent the mean ± SD of three independent experiments performed in triplicate; # *p* < 0.05 and ** *p* < 0.01.

Fascaplysin has been reported to exert an anti-cancer effect through the inhibition of CDK4 activity [6,7,8]. Therefore, we tested whether the inhibition of the CDK4-RB axis by fascaplysin is a major mechanism for its anti-cancer effect. First, we found that fascaplysin rapidly decreased cell viability in multiple types of cancer cells (Figure S1A). Interestingly, Figure 1A shows that fascaplysin sensitively suppressed A375 malignant melanoma cell growth compared to that when two different selective CDK4 inhibitors, PD0332991 and LY2835219, were used. To determine the effective concentration of CDK4 inhibitors including fascaplysin on RB inhibition, we measured phosphorylated RB levels in CDK4 inhibitors in a dose-dependent manner. Figure 1C shows that PD0332991 or LY2835219 more effectively suppressed RB phosphorylation at approximately a five-fold lower concentration than fascaplysin. Unlike that observed with fascaplysin on RB suppression, decreased cell viability was observed to be greater than four-fold when compared to that observed with PD0332991 or LY2835219 at the same concentration (Figure 1B). Moreover, fascaplysin dramatically decreased cell viability in RB-null NCI-H596 lung cancer cells (Figure 1D). To compare the pro-apoptotic effect of CDK4 inhibitors, we quantified apoptotic cell numbers in the absence or presence of CDK4 inhibitors. Fascaplysin, but not PD0332991 and LY2835219, increased activation of caspase-9, -3, and PARP (Figure 1E and Figure S1B). In addition, the pan-caspases inhibitor *Z*-VAD-FMK significantly rescued fascaplysin-induced apoptosis, suggesting that caspase-mediated apoptosis is more critical than the inhibition of CDK4-mediated cell cycle progression to cancer clearance by fascaplysin (Figure 1F and Figure S1C). These results suggest that fascaplysin induced cancer cell death independently of the CDK4-RB axis, and other target molecules that are inhibited by fascaplysin could be involved in the anti-cancer effects of fascaplysin.

### 2.2. Survivin Is Involved in Fascaplysin-Induced Apoptosis

**Figure 2:**
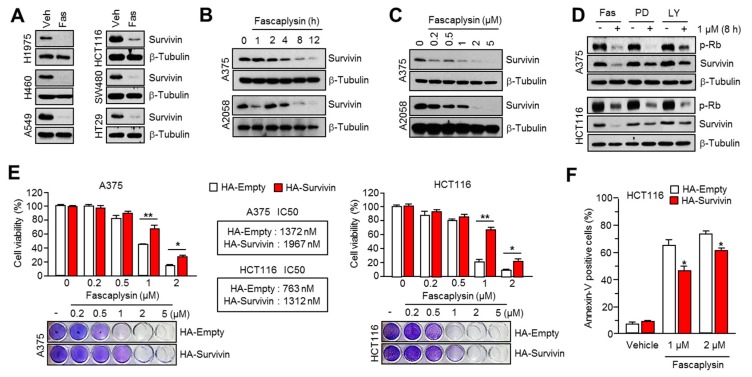
Fascaplysin induced apoptosis by suppressing survivin expression. (**A**) Multiple types of cancer cells were incubated with 1 µM of fascaplysin for 12 h, and then the survivin protein was measured by western blotting; (**B**,**C**) A375 and A2058 cells were treated with fascaplysin in a time- or dose-dependent manner as indicated. The levels of survivin were measured by Western blotting; (**D**) A375 and HCT116 cells were incubated with 1 µM of CDK4 inhibitors for 8 h; (**E**) The cell viability was measured in A375 or HCT116 cells that were overexpressing an empty vector or HA-tagged survivin upon fascaplysin treatment as indicated. Crystal violet staining images are shown. Values represent the mean ± SD of three independent experiments performed in triplicate; * *p* < 0.05 and ** *p* < 0.01; (**F**) HCT116 cells overexpressing an empty vector or HA-tagged survivin were incubated for 48 h in the absence or presence of 1 or 2 µM of fascaplysin. After annexin-V staining, the population of cells was determined by FACS analysis. Values represent mean ± SD of three independent experiments performed in triplicate; * *p* < 0.05.

Survivin, which is overexpressed in multiple types of cancer but not in terminally-differentiated normal tissues, is well studied as an attractive candidate for cancer therapy because of its inhibitory function against extrinsic or intrinsic apoptotic pathways [10]. Fascaplysin increases apoptosis through the activation of caspases (Figure 1), which suggests the suppression of anti-apoptotic factors. To test this possibility, we first measured survivin protein levels in several solid cancer cells in the absence or presence of fascaplysin. Figure 2A shows that survivin level was decreased in fascaplysin-treated cancer cells. Additionally, fascaplysin dramatically suppressed survivin protein levels, but not mRNA, in a time- and dose-dependent manner (Figure 2B,C and Figure S2A). The comparison with other CDK4 inhibitors on survivin expression shows that fascaplysin, but not PD0332991 and LY2835219, specifically decreased survivin, indicating that fascaplysin decreases survivin independently of CDK4 inhibition (Figure 2D). To evaluate whether survivin mediates fascaplysin-induced apoptosis, we generated A375 or HCT116 cells overexpressing a HA-tagged survivin construct (Figure S2B). These cells were resistant to cell growth inhibition (Figure 2E) and apoptosis (Figure 2F and Figure S2C) by fascaplysin treatment. These results indicated that fascaplysin decreased cell viability and increased apoptosis by suppressing survivin expression.

### 2.3. Fascaplysin Downregulates De Novo Synthesis of Survivin Protein by Inhibiting Cap-Dependent Translation Controlled by 4EBP1

**Figure 3:**
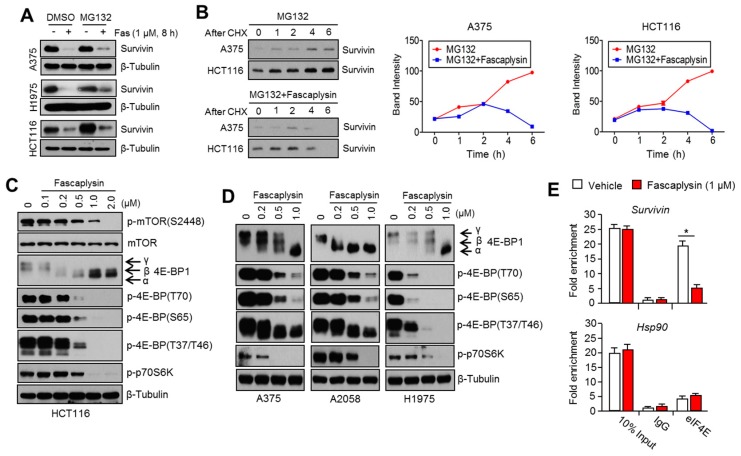
Fascaplysin inhibited 4EBP1-mediated cap-dependent de novo protein synthesis. (**A**) The cells were pre-treated with 20 µM of MG132 for 1 h, and then further incubated in the absence or presence of 1 µM of fascaplysin for 8 h. The protein levels of survivin were measured by Western blotting; (**B**) Protein synthesis in A375 or HCT116 cells was blocked and synchronized using 100 nM of cycloheximide (CHX) for 12 h. After removing CHX, the cells were further incubated with 20 µM of MG132 in the absence or presence of 1 µM of fascaplysin for the indicated time prior to lysis. Relative band intensities are shown; (**C**,**D**) The cells were incubated with various concentrations of fascaplysin for 6 h, and then the levels of the indicated proteins were measured by Western blotting; (**E**) Cytoplasmic proteins (1 mg) from fascaplysin-treated A375 cells were incubated with 1 μg of the indicated antibodies. The precipitated proteins–mRNA complexes were treated with proteinase K to elute mRNAs, and then the indicated mRNA levels were measured by quantitative real time-PCR. Values represent mean ± SD of two independent experiments performed in triplicate; * *p* < 0.05.

Since fascaplysin does not affect the expression of survivin mRNA (Figure S2A), we hypothesized that fascaplysin may enhance ubiquitination-mediated degradation or attenuate de novo protein synthesis of survivin. First, we found that the 26S proteasome inhibitor MG132 did not prevent survivin suppression upon fascaplysin treatment in three different cell lines (Figure 3A). Thus, we analyzed the de novo protein synthesis of survivin. Figure 3B shows that fascaplysin significantly attenuated the accumulation of survivin protein after its release by blocking protein synthesis as a result of cycloheximide (CHX) pre-treatment in A375 and HCT116 cells. This result indicates that fascaplysin suppresses survivin expression through the inhibition of protein synthesis. Since protein synthesis of several oncoproteins including survivin, HIF-1α, and cyclin D1 are tightly regulated by cap-dependent translation through the mTOR-4EBP1-p70S6K1 pathway [18,19], we further investigated whether fascaplysin can inhibit the mTOR-4EBP1-p70S6K1 pathway. Figure 3C,D show that fascaplysin attenuated the phosphorylation status of mTOR, 4EBP1, and p70S6K1 in a dose-dependent manner in four different cancer cell lines. Moreover, we found that survivin mRNA levels, which bound to eIF4E, were decreased upon fascaplysin treatment (Figure 3E), and this result was consistent with the fact that non-phosphorylated 4EBP1 in its active form interferes with the assembly of eIF4E on the 5’-UTR region of survivin mRNA. In addition, HIF-1α, which is fine-tuned by cap-dependent translation, and its target genes were also downregulated by fascaplysin under hypoxic conditions or MG132 treatment (Figure S3A,B). We measured c-myc and cyclin D1, which are known as cap-dependent translation sensitive mRNAs, and strong reduction of c-myc and cyclin D1 was observed upon fascaplysin treatment (Figure S3C). These results suggest that fascaplysin suppresses the expression of survivin and HIF-1α by attenuating cap-dependent translation that is being regulated by the mTOR-4EBP1-p70S6K1 pathway.

### 2.4. Anti-Angiogenic and Pro-Apoptotic Effects of Fascaplysin In Vivo

**Figure 4:**
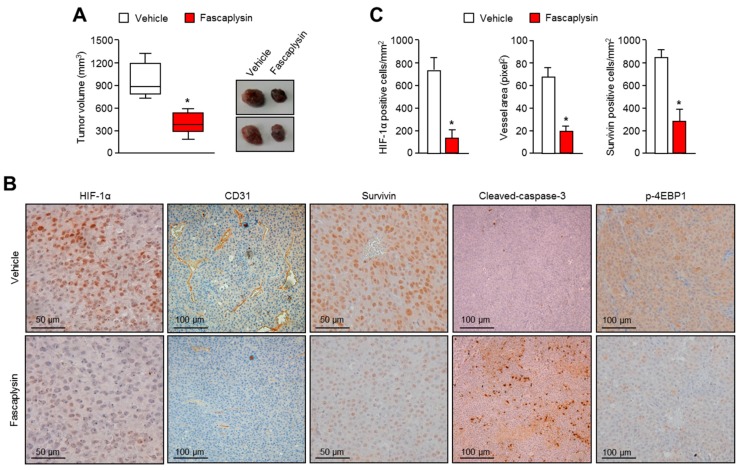
Effects of fascaplysin on angiogenesis and apoptosis in the xenografted tumor models. (**A**) Three weeks after injection, mice were injected daily with fascaplysin or dimethyl sulfoxide (DMSO) and then the tumor volumes were measured. In the box plots, the final tumor volumes are shown (*n* = 6). The whiskers in the box plots represent the maximum and the minimum value; * *p* < 0.05; (**B**) Tumors were fixed and embedded into paraffin blocks and then cut into 6 µm sections. Tumor tissues were immunostained with the indicated antibodies. Immunostained slides were visualized with the avidin-biotin-horseradish peroxidase method using diaminobenzidine as the chromogen; (**C**) Three sections of each tumor (five fields per section) were reviewed for histologic assessments. HIF-1α and survivin protein levels were determined by counting of immunopositive cells. The area of each CD31-positive vessel was determined in units of pixel2 using the Image J software. Vascular area per mm^2^ was calculated by dividing the sum of the vascular areas by the total tissue area; * *p* < 0.05.

Next, we investigated the anti-tumor effects of fascaplysin using the human malignant melanoma A375 cell-injected xenograft model. Consistent with the anti-cancer effects in vitro (Figure 1), end-point tumor volumes were significantly reduced upon fascaplysin treatment (Figure 4A). We have not observed any adverse effects of fascaplysin like reduction of body weight or abnormal behavior, and another study has also shown no-cytotoxicity of fascaplysin at 10 mg/kg, which is much higher in concentration compared with that of our study [21]. Immunohistochemical analyses showed that the levels of tumor angiogenic factors (HIF-1α and CD31), anti-apoptotic factor (survivin), and de novo protein synthesis regulators (p-4EBP1) were dramatically decreased upon fascaplysin treatment Figure 4B). In addition, one of the apoptotic markers, cleaved-caspase-3, was significantly increased in fascaplysin-treated tumor tissues (Figure 4B). Figure 4C shows that the numbers of HIF-1α- and survivin-positive cancer cells and blood vessels were decreased by fascaplysin treatment. These results indicated that fascaplysin exerts anti-angiogenic and pro-apoptotic effects in vivo.

### 2.5. Kinase Activity of TRKA and VEGFR2 Was Inhibited by Fascaplysin through Asp-Phe-Gly (DFG)-Out Non-Competitive Inhibition

**Figure 5:**
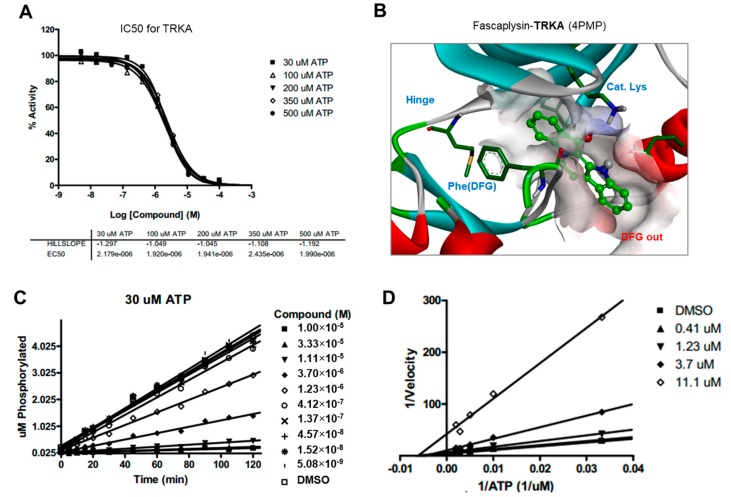
ATP competition studies over TRKA. The inhibition of TRKA by fascaplysin was not time-dependent and was noncompetitive with respect to ATP. (**A**) IC50 curves for TRKA at different ATP concentrations; (**B**) Calculated binding modes of fascaplysin in TRKA; (**C**) Progress curves for TRKA/fascaplysin; (**D**) Lineweaver-Burk plot for TRKA/fascaplysin.

**Figure 6:**
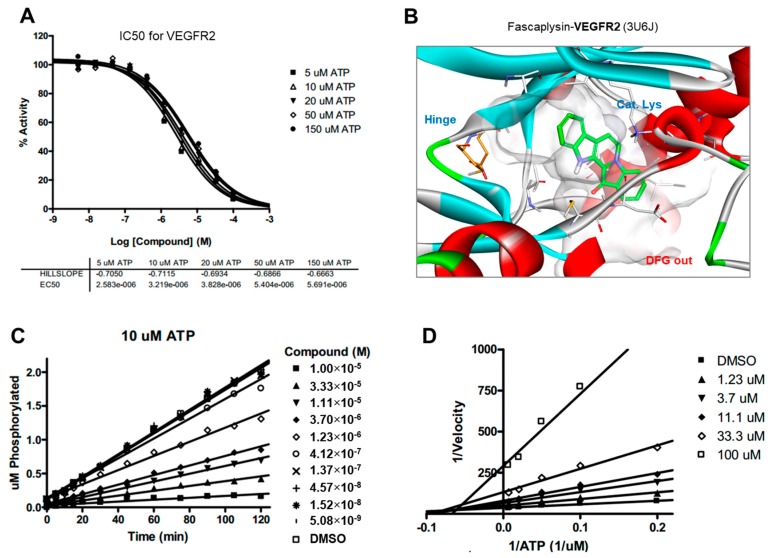
ATP competition studies over VEGFR2. The inhibition of VEGFR2 by fascaplysin was not time-dependent and was noncompetitive with respect to ATP. (**A**) IC50 curves for VEGFR2 at different ATP concentrations; (**B**) Calculated binding modes of fascaplysin in VEGFR2; (**C**) Progress curves for VEGFR2/fascaplysin; (**D**) Lineweaver-Burk plot for VEGFR2/fascaplysin.

To elucidate the inhibitory activity towards the mTOR-4EBP1-p70S6K1 signaling pathway, fascaplysin was subjected to a high-throughput kinase binding assay. Fascaplysin has been revealed as a critical inhibitor of cancer-associated kinases, including VEGFR3, VEGFR2, and TRKA with IC50 values in the range of 2–3 µM (Figure S4). To understand the binding mode of action in the ATP-binding site, we performed ATP competition evaluation studies. When the slopes of enzyme activities relative to that of the dimethyl sulfoxide (DMSO) control at each ATP concentration were plotted against the fascaplysin concentration, the IC50 values did not change significantly when ATP concentration was increased, as expected for normal noncompetitive inhibitors (Figure 5A and Figure 6A). In addition, as shown in the Lineweaver-Burk plots, all lines converged on the X-axis in the double-reciprocal plot (Figure 5D and Figure 6D), suggesting that fascaplysin is noncompetitive with respect to ATP against TRKA and VEGFR2. The progress curves were linear, regardless of the concentration of fascaplysin (Figure 5C and Figure 6C), indicating that the inhibition over TRKA and VEGFR2 by fascaplysin was not time-dependent. To identify the key intermolecular interactions required for tight binding of fascaplysin, docking simulation studies were performed using the X-ray crystal structure of TRKA (PDB entry: 4PMP) and VEGFR2 (PDB entry: 3U5J) as shown in Figure 5B and Figure 6B. Binding mode analyses and docking simulations further supported a noncompetitive inhibition mechanism for TRKA and VEGFR2 inhibition of fascaplysin by stabilizing the DFG-out inactive conformation. Since we found that VEGFR3 may be a potential target of fascaplysin by high-throughput kinase bind assay, we evaluated whether pharmacological inhibition of VEGFR3 could mimic fascaplysin on suppression of 4EBP1 phosphorylation and cell viability. Here, we found that selective inhibitor of VEGFR3 and MAZ51 is sufficient to suppress 4EBP1 phosphorylation, as well as survivin expression (Figure S4B,C). In addition, decreased cell viability in A375 and A2058 melanoma cells was observed upon MAZ51 treatment (Figure S4D). These results indicate that VEGFR3, VEGFR2, and TRKA, as potential fascaplysin targets, may be associated with anti-cancer effect of fascaplysin.

### 2.6. Fascaplysin Sensitizes the Anti-Cancer Effect of TRAIL

**Figure 7:**
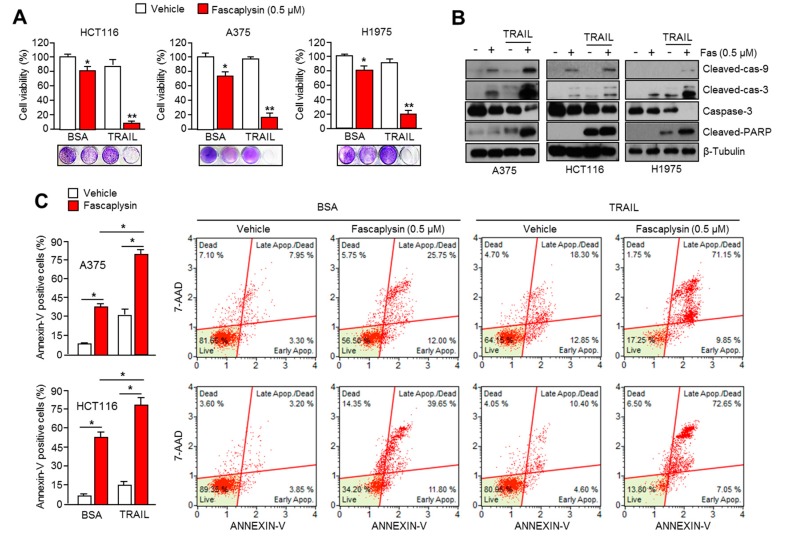
Fascaplysin sensitizes the anti-cancer effect of TRAIL. (**A**) Cell viability was measured in HCT116, A375, and H1975 under a combination of fascaplysin and TRAIL. Cells were incubated with 0.5 µM of fascaplysin and 20 ng/mL of TRAIL for 24 h. Values represent mean ± SD of three independent experiments performed in triplicate; * *p* < 0.05 and ** *p* < 0.01; (**B**) The cells were treated with a combination of 0.5 µM of fascaplysin and 20 ng/mL of TRAIL for 12 h, and then cleaved-caspase-9, -3, and PARP were determined by Western blotting; (**C**) A375 and HCT116 cells were incubated with a combination of 0.5 µM of fascaplysin and 20 ng/mL of TRAIL for 24 h. After annexin-V staining, the population of cells was determined by FACS analysis. Values represent mean ± SD of three independent experiments was performed in triplicate; * *p* < 0.05.

Since small molecules targeting survivin expression are sensitized to TRAIL-mediated apoptosis in cancer cells [13,14,15,16,17], we investigated whether fascaplysin enhances the anti-cancer effect of TRAIL in multiple types of cancer cells. Figure 7A shows that 0.5 µM of fascaplysin strongly decreased cell viability in the presence of TRAIL in HCT116 (colorectal), A375 (malignant melanoma), and H1975 (lung) cancer cells. Moreover, activation of caspase-9, -3, and PARP was further increased by a combination of fascaplysin and TRAIL (Figure 7B). Consistently, apoptotic cells were synergistically increased by the fascaplysin and TRAIL combination (Figure 7C and Figure S5). These results indicated that fascaplysin is a potential drug for improving the anti-cancer effect of TRAIL, which has not been very successful in clinical trials because of its low efficacy for tumor clearance.

## 3. Discussion

Previous studies have shown the anti-cancer effect of fascaplysin through the suppression of CDK4 and cell cycle progression in various cancer cells [6,7,8,22], but the precise molecular mechanism by which fascaplysin attenuates cancer cell growth remained unexplored. Here, for the first time, we examined the dependence of the CDK4-RB axis in the anti-cancer effect of fascaplysin. Interestingly, our results revealed that fascaplysin rapidly decreased cell viability and increased apoptosis when compared to other CDK4 inhibitors, such as PD0332991 and LY2835219, in various cancer cells. Moreover, the anti-cancer effect of fascaplysin was also observed in RB-null NCI-H596 lung cancer cells. These results indicated that key endogenous targets of fascaplysin, which might be critical factors for tumor growth, have not yet been explored. 

Recently, Kumar et al. have reported that fascaplysin attenuated PI3K-AKT-mTOR signaling in HL-60 cells [9]; however, the molecular mechanism by which fascaplysin inhibits the PI3K-AKT-mTOR pathway has not been explored. In the present study, we performed a high-throughput kinase binding assay and identified that multiple types of cancer-related kinases, such as VEGFR3, VEGFR2, and TRKA, were inhibited by fascaplysin at concentrations lower than 3 µM. This finding suggests that the PI3K-AKT-mTOR pathway might be indirectly inhibited by fascaplysin via the suppression of the VEGFR and TRKA pathways. Further studies whether fascaplysin attenuates autophosphorylation or activation of VEGFR2 or TRKA that stimulate signal cascades related to cancer cell growth and proliferation would strengthen our finding as well. Similarly, the high-throughput kinase binding assay revealed that PI3K, AKT, and mTOR might not be directly affected by fascaplysin. Given the fact that VEGFRs and TRKs are emerging potential targets for cancer-targeted therapies, these findings provide a possibility that fascaplysin can be a valuable candidate for the development of cancer-targeting drugs. 

Drug and protein docking simulations were analyzed to obtain energetic and structural insight into the binding modes between fascaplysin and TRKA or fascaplysin and VEGFR2. In the calculated TRKA–fascaplysin complex shown in Figure 5B, fascaplysin appears to be in close contact with residues Val573, Leu657, Ile572, Phe646, Leu563, and Phe669, all of which are close to the DFG motif. It is noted that the C=O and NH bond of fascaplysin form two hydrogen bonds with a catalytic lysine (Lys544) and Glu560 of the α-C helix, respectively. Fascaplysin may be further stabilized via an attractive charge interaction between the endocyclic ammonium cation with the side chain of Asp668 and via a π-stacking interaction with the benzyl side chain of the gatekeeper Phe589. Fascaplysin also displayed high affinity towards VEGFR2 and VEGFR3. This binding affinity was found to be similar to that of TRKA, in that fascaplysin is stabilized by Van der Waals interactions with Ile892, Leu1035, Thr916, and Val914 in the DFG-out conformation. The carbonyl group of fascaplysin forms a hydrogen bond with Asp1046 and an attractive charge interaction of the endocyclic ammonium cation with the side chain of Glu885 in the α-C helix, respectively. These findings may be helpful for understanding the design of fascaplysin-based derivatives as potential anticancer drugs.

Although the precise molecular mechanism is not clearly understood, previous studies have revealed that fascaplysin attenuates tumor angiogenesis in vitro and in vivo [21,23]. In this study, a drug–protein docking simulation and several biochemical assays suggest that fascaplysin inhibits VEGFR2, which is a potential target for cancer treatment. Moreover, the administration of fascaplysin strongly reduced the accumulation of HIF-1α, which is a key transcriptional factor for regulating tumor angiogenesis in hypoxic tumor in vitro and in vivo. Consistently, histological analysis revealed that fascaplysin decreased blood vessels in xenografted tumor tissues. These findings suggest that fascaplysin attenuates tumor angiogenesis via the suppression of HIF-1α and VEGFR2.

Given the fact that cap-dependent translation regulated by the mTOR-4EBP1-eIF4E axis fine-tunes the expression of several oncoproteins, such as survivin, c-myc, cyclin D1, VEGF, and HIF-1α, all of which are called eIF4E-sensitive mRNAs, at the post-transcriptional level [18,19], therapeutic suppression of cap-dependent translation has been considered as a valuable strategy for cancer-targeted therapy. Indeed, several reports have shown that small molecules targeting the mTOR-4EBP1-eIF4E axis reduces cancer growth in many experimental models [18,19,24]. Herein, we found that fascaplysin suppresses the phosphorylation of mTOR, 4EBP1, and p70S6K1, all of which are pivotal signaling molecules for the cap-dependent translation machinery. Additionally, we showed that fascaplysin attenuated survivin protein synthesis by dissociating eIF4E from survivin mRNA. These findings reveal that fascaplysin could be considered as a candidate for the development of anti-cancer drugs targeting cap-dependent translation machinery.

Although it is clear that TRAIL is a promising agent for cancer-targeted therapy because of its tumor selectivity, its low efficacy has been observed in many tumors due to the resistance to TRAIL-based therapies [17,25]. Many reports have shown that several chemopreventive agents and anti-cancer drugs, including PPARγ ligands, resveratrol, aspirin, HDAC inhibitors, and proteasome inhibitors could improve the anti-cancer efficacy of TRAIL-based therapies [13,14,15,16]. The importance of survivin suppression in cancer treatment has also been demonstrated by its ability to overcome resistance to TRAIL-based cancer therapy. Indeed, a previous report has shown that the pharmacological inhibition or genetic silencing of survivin sensitizes tumors to TRAIL-based cancer therapy [16]. Consistently, fascaplysin suppresses survivin expression, and here we found that TRAIL-induced cancer cell death was significantly increased by fascaplysin in malignant melanoma, colorectal, and lung cancer cells. These results suggest that fascaplysin might be a potential agent for overcoming resistance to TRAIL-based cancer therapies. 

## 4. Materials and Methods

### 4.1. Reagents and Antibodies

Fascaplysin, PD0332991, and LY2835219 were purchased from Selleck Chemicals (Houston, TX, USA). *Z*-VAD-FMK, MG132, MAZ51, and cycloheximide were purchased from Sigma Aldrich (St. Louis, MO, USA). Recombinant TRAIL was purchased from PeproTech (Rocky Hill, NJ, USA). For Western blotting, antibodies recognizing phospho-mTOR (CST-5536), mTOR (CST-2983), phospho-4EBP1T70(sc-18092), phospho-4EBP1S65(sc-18091), phospho-4EBP1T37/T46(CST-2855), 4EBP1 (CST-9452), phospho-p70S6K1 (CST-9234), phospho-RB (CST-8516), cleaved-caspase-9 (CST-9505), cleaved-caspase-3 (CST-9664), cleaved-PARP (CST-5625), c-myc (sc-40), cyclin D1 (sc-8396), and β-tubulin (sc-9104) were purchased from Cell Signaling Technology (Danvers, MA, USA) and Santa Cruz Biotechnology (Dallas, TX, USA). Antibodies recognizing survivin (AF886), and HIF-1α (ab1) were purchased from R&D systems, Inc. (Minneapolis, MN, USA) and Abcam (Cambridge, MA, USA).

### 4.2. Cell Culture and Generation of Stable Cell Lines

All cancer cell lines were maintained in Dulbecco’s Modified Eagle’s medium (DMEM) containing 10% fetal bovine serum and 25 mM glucose. The human survivin–expressed lentiviral vector, pLenti-GIII-CMV-Survivin (LV089651), was purchased from Applied Biological Materials Inc. (Richmond, BC, Canada). The lentiviral and packaging vectors were co-transfected into HEK293T cells, the lentiviral particles were collected after 72 h of transfection. The lentiviral particles were infected into A375 or HCT116 cells, and survivin-overexpressing cells were selected by 2 µg/mL of puromycin (Invitrogen, Carlsbad, CA, USA) for six days. 

### 4.3. Western Blotting

The cells were lysed with RIPA buffer containing 50 mM Tris-HCl (pH 7.9), 150 mM NaCl, 1% IGEPAL, 10 mM NaF, 0.1 mM EDTA, and a protease inhibitor cocktail. Protein samples were loaded onto sodium dodecyl sulfate-polyacrylamide gel electrophoresis (SDS-PAGE), and transferred onto a PVDF membrane (Millipore, Billerica, MA, USA). Afterwards, primary antibodies were added to the membrane (1:1000–1:5000) at 4 °C for overnight. The second day, the membrane was reacted with secondary antibodies for 1 h at room temperature. The protein band appeared by adding substrate (Enhanced Chemiluminescence (ECL) Prime kit, GE healthcare, Pittsburgh, PA, USA).

### 4.4. RNA-Binding Protein Immunoprecipitation (RIP) Assay

As previously described [26], cytoplasmic extracts were prepared using a lysis buffer containing IGEPAL (0.5%), KCl (120 mM), Tris-HCl (pH 7.9, 50 mM), EDTA (2 mM), RNase inhibitor (100 U/mL), and a protease inhibitor cocktail. Cell lysates were incubated with 1 µg/mL of antibody against eIF4E or normal IgG at 4 °C overnight, and then eIF4E-bound RNAs were measured by quantitative real time (RT)-PCR. 

### 4.5. Quantitative Real Time-PCR

Total RNA was isolated from cells using TRIzol (Invitrogen), and RNA (2 µg) was reverse-transcribed—by a high-capacity cDNA reverse transcription kit (Applied Biosystems, Foster city, CA, USA). Quantitative real time PCR was performed by equal amounts of cDNA mixed with SYBR Green PCR Master Mix (Applied Biosystems), and the following primers (5′–3′). CTTTCTCCGCAGTTTCCTCA and TTGGTGAATTTTTGAAACTGGA for survivin; GGCATTGATGACTCCAGTGTT and ATGGAGCCCAGCAGCAA for GLUT1; AGCTGCGCTGATAGACATCC and CTACCTCCACCATGCCAAGT for VEGF; GGAGATCCATCATCTCTCCC and GGCCTGTGCCATCAGTATCT for LDHA; ATTTTCCTCAAAGGAACGCC and CAACAGAGGTGTTTACCCCC for PDK1.

### 4.6. Tumor Xenograft Assay and Immunohistochemistry

As previously described [27], A375 malignant melanoma cells (1 × 10^6^) were subcutaneously injected into the flank of Balb/c-nude mice in 100 µL of FBS-free media. Three weeks after cell injection, mice were injected daily with fascaplysin (1 mg/kg) or DMSO by intraperitoneal injection in the control group for 10 days. Tumor volumes were measured with calipers and calculated using the following equation: volume = ab^2^/2, where a is the maximal width and b is the maximal orthogonal width. The animal experiments were conducted and managed in accordance with the guidelines of the Konkuk University Institutional Animal Care and Use Committee (KU15072-1; approved at 21 September 2015). Immunohistochemical staining was performed as previously described [28]. Tumor tissues were fixed with 4% paraformaldehyde, embedded in paraffin, and cut into 6-µM sections. They were deparaffinized and rehydrated, and then autoclaved for 10 min in 10 mM sodium citrate (pH 6.0) to retrieve the target antigens. These sections were then incubated with anti-HIF-1α (1:50; provided by Jong-Wan Park in Seoul National University), anti-CD31 (1:50; ab28364), anti-survivin (1:100; sc-17779), anti-cleaved-caspase-3 (1:50; CST-9991), or anti-phospho-4EBP1 (1:50; CST-2855) overnight at 4 °C. After the primary antibody reaction, sections were further incubated with biotinylated secondary antibodies. Finally, immunocomplexes were visualized using diaminobenzidine (DAB) staining. 

### 4.7. Docking Simulation Studies

Docking simulation studies of TRKA and VEGFR2 in complex with fascaplysin were conducted with the Discovery Studio 4.5 (San Diego, CA, USA, version 4.5) using the all-atom model prepared by adding the missing atoms to the original X-ray crystal structure. The three-dimensional atomic coordinates required for the docking study were prepared from the X-ray crystal structure of TRKA (PDB entry: 4PMP) and VEGFR2 (PDB entry: 3U5J) in complex with fascaplysin. These uniquely defined potential grids for TRKA and VEGFR2 were then used in common for docking simulations. To construct the all-atom model for TRKA and VEGFR2, hydrogen atoms were added to the individual protein atoms after removing the crystallographic water molecules. The ionization states of the titratable residues (Asp, Glu, His, and Lys) were determined prior to building the model according to the hydrogen bond formation patterns revealed in the original X-ray structures.

### 4.8. Kinase Inhibition Assay

The inhibitory activities of fascaplysin with respect to TRKA and VEGFR2 were measured by means of radiometric kinase assays ([γ-^33^P]ATP) (Reaction Biology Corp. Malvern, PA, USA). The enzymatic activity of TRKA and VEGFR2 was monitored using 20 µM of peptide substrate, dissolved in the freshly-prepared reaction buffer (20 mM HEPES (pH 7.5), 10 mM MgCl_2_, 1 mM EGTA, 0.02% BRIJ-35, 0.02 mg/mL BSA, 0.1 mM Na_3_VO_4_, 2 mM DTT, 1% DMSO). Each putative fascaplysin was dissolved in 100% DMSO at the specific concentration and diluted in a serial manner with epMotion 5070 in DMSO. Either TRKA (recombinant human cytoplasmic domain (aa 441–796, accession number NP_002520), C-terminal His-tagged, expressed in insect cells. Molecular weight (Mw) = 42.8 kDa) or VEGFR2 (recombinant human catalytic domain (aa 789–1356; accession number: NM_002244), C-terminal His-tagged, expressed in insect cells. Activated in vitro via autophosphorylation; Mw = 68.4 kDa) were added into the reaction buffer including 20 µM of peptide substrate. After delivering the candidate inhibitor dissolved in DMSO into the kinase reaction mixture by acoustic technology (Echo 550; nanoliter range), and the reaction mixture was incubated for 20 min at room temperature. To initiate the enzymatic reaction, ^33^P-ATP with specific activity of 10 µCi/µL was delivered into the reaction mixture to reach the final ATP concentration of 10 µM. Radioactivity was then monitored by the filter binding method after the incubation of reaction mixture for 2 h at room temperature. At given concentrations of fascaplysin, biochemical potency was measured by the percent of kinase activity remaining with respect to the vehicle (dimethyl sulfoxide) reaction. Curve fits and IC_50_ values were then obtained using the PRISM program (GraphPad Software, La Jolla, CA, USA, version 5.01). The kinase assays were performed at room temperature.

### 4.9. Measurement of Cell Viability

To measure cell viability, cells were seeded onto 12- or 24-well tissue culture dishes and incubated for one, two, or three days in the absence or presence of fascaplysin. After incubation, cultured cells were washed with phosphate-buffered saline (PBS) and then stained with 0.5% crystal violet. After 20 min, the stained cells were dissolved with 1% SDS solution for three times at room temperature, and then the optical density was read at 570 nm using microplate reader (BioTek, Winooski, VT, USA).

### 4.10. Apoptosis Assays

For the apoptosis assay, cells were seeded at a density 1 × 10^5^ cells/well onto a six-well cell culture plate and treated with fascaplysin (0.5, 1 µM), TRAIL (20 ng/mL), and *Z*-VAD-FMK (20 µM) at various concentrations. The adherent cells were collected and washed with cold PBS, and then centrifuged at 2000 rpm for 2 min at room temperature. The pellet was resuspended in 1 mL of medium, and then 100 µL of the suspension was transferred into a fresh tube. Then, 100 µL of Muse™ Annexin V and Dead Cell kit reagents (EMD Millipore, Billerica, MA, USA) were added to each tube and incubated for 20 min at room temperature in the dark. The stained samples were analyzed using a Mini Flow Cytometry Muse™ Cell Analyzer (EMD Millipore).

### 4.11. Statistical Analysis

For analyze between two experimental data, the unpaired Student’s *t*-test was used. For multiple comparisons, one-way ANOVA with Tukey post-test was performed. Data are shown as means ± standard deviations (SD). If the *p*-value was under 0.05, the data was regarded as statistically significant.

## 5. Conclusions

Taken together, our results provide several advantages of fascaplysin with newly-identified modes of action for the development of anti-cancer drugs: (1) fascaplysin exerts anti-angiogenic effects via the suppression of VEGFR3, VEGFR2, and HIF-1α; (2) fascaplysin exerts a synergistic effect on TRAIL-based cancer therapies by suppressing survivin expression; and (3) fascaplysin directly suppresses several cancer-related RTKs, such as VEGFR2 and TRKA via DFG-out non-competitive inhibition.

## Supplementary Materials

Supplementary materials can be found at www.mdpi.com/1422-0067/18/10/2074/s1.

## Abbreviations

CDK4	Cyclin-dependent kinase 4
RB	Retinoblastoma
TRKA	Tropomyosin-related kinase A
VEGFR2	Vascular endothelial growth factor receptor 2
HIF-1α	Hypoxia-inducible factor-1α
mTOR	Mammalian target of rapamycin
4EBP1	Eukaryotic translation initiation factor 4E-binding protein 1
RTK	Receptor tyrosine kinase
EGFR	Epidermal growth factor receptor
PDGFR	Platelet-derived growth factor receptor
TRAIL	TNF-related apoptosis-inducing ligand
