# Supplementary material for: Fascaplysin Exerts Anti-Cancer Effects through the Downregulation of Survivin and HIF-1α and Inhibition of VEGFR2 and TRKA
Source: Int J Mol Sci. 2017 Sep 29;18(10):2074. doi: 10.3390/ijms18102074 (PMC5666756; doi:10.3390/ijms18102074)
Supplement: Supplementary file 1 [file ijms-18-02074-s001.pdf]

Supplementary Material

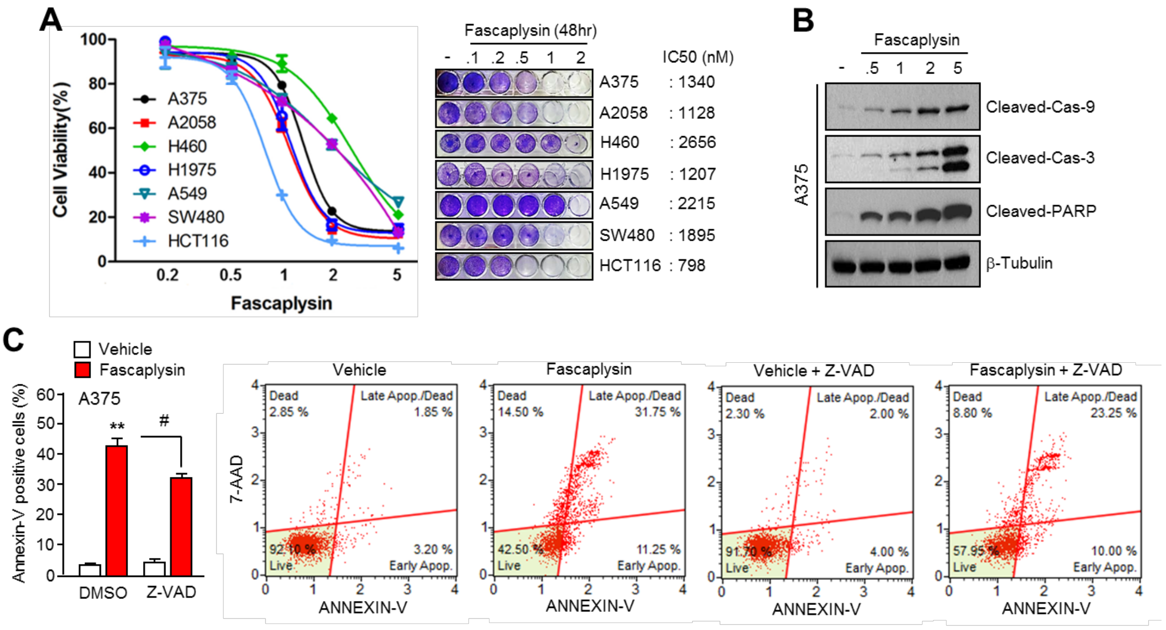

Figure S1. Fascaplysin rapidly induced apoptosis in multiple types of cancer cells. (**A**) The growth inhibition by fascaplysin in various cancer cells. Cells were incubated in the absence or presence of fascaplysin for 48 h, and then cell viability was measured by crystal violet staining. Crystal violet staining images and IC50 values are shown. Values represent mean ± SD of three independent experiments performed in triplicate; (**B**) A375 melanoma cells were treated with fascaplysin for 24 h, and then cleaved-caspase-9, -3, and PARP were determined by western blotting; (**C**) A375 melanoma cells were incubated with 1 µM of fascaplysin for 48 h in the absence or presence of the pan-caspase inhibitor Z-VAD-FMK. After annexin-V staining, the population of cells was determined by FACS analysis. Values represent mean ± SD of three independent experiments performed in triplicate; $^{\#}$ $p < 0.05$ and ** $p < 0.01$.

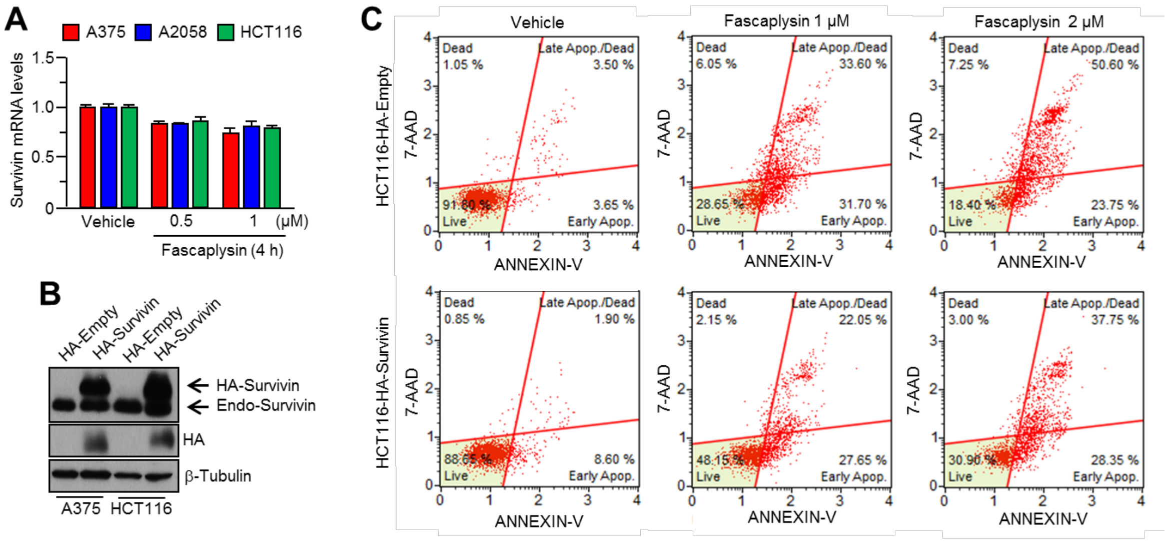

Figure S2. Survivin overexpressing HCT116 cells are resistant to fascaplysin-induced apoptosis. (**A**) Fascaplysin does not affect survivin mRNA expression. The cells were incubated in the absence or presence of fascaplysin for 4 h, and then mRNA levels were measured using quantitative real time (RT)-PCR. Values represent mean ± SD of three independent experiments performed in duplicate; (**B**) HA-tagged survivin protein levels were shown; (**C**) HCT116 cells overexpressing an empty vector or HA-tagged survivin were incubated for 48 h in the absence or presence of 1 or 2 µM of fascaplysin. After annexin-V staining, the population of cells was determined by FACS analysis.

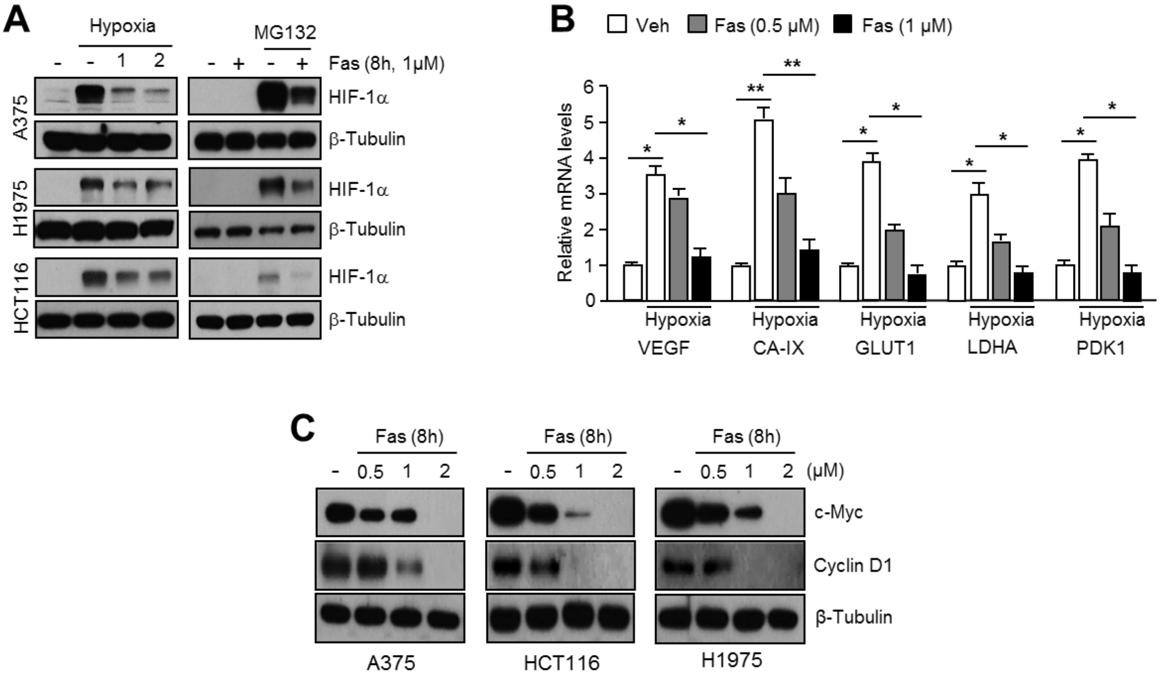

Figure S3. Fascaplysin decreases HIF-1α protein and its target gene expression. (**A**) HIF-1α decreased in fascaplysin-treated cancer cells. The cells were treated with fascaplysin for 8 h in normoxic or hypoxic condition (left). The cells were treated with fascaplysin for 8 h in the absence or presence of 26S proteasome inhibitor, MG132 (right). HIF-1α protein levels were measured using western blotting; (**B**) Fascaplysin suppresses HIF-1α target gene expression under hypoxia. A375 malanoma cells were incubated in the absence or presence of fascaplysin for 24 h, and then mRNA levels were measured using quantitative real time (RT)-PCR. Values represent mean ± SD of three independent experiments performed in duplicate; $^{\#}$ $p < 0.05$ and $^{**}p < 0.01$; (**C**) Fascaplysin decreases the cap-dependent translation sensitive mRNA, such as c-myc and cyclin D1. Protein levels were measured using western blotting.

A

#	target	IC_{50} (μM)[a]
1	AKT1	5.68
2	AKT2	3.58
3	AKT3	4.14
4	CDK2	3.54
5	CDK4-cyclinD1	8.44
6	c-KIT	20.3
7	MKK4	31.3
8	MKK6	17.9
9	MKK7	7.12
10	RON/MST1R	5.47
11	PDGFRa	8.00
12	PDGFRb	4.87
13	PDPK1	8.73
14	TRKA	2.51
15	TRKB	4.02
16	TRKC	4.18
17	AXL	5.83
18	RET	8.07
19	KDR/VEGFR2	2.87
20	FLT1/VEGFR1	3.20
21	FLT4/VEGFR3	1.14

[a]Reaction Biology Corp.

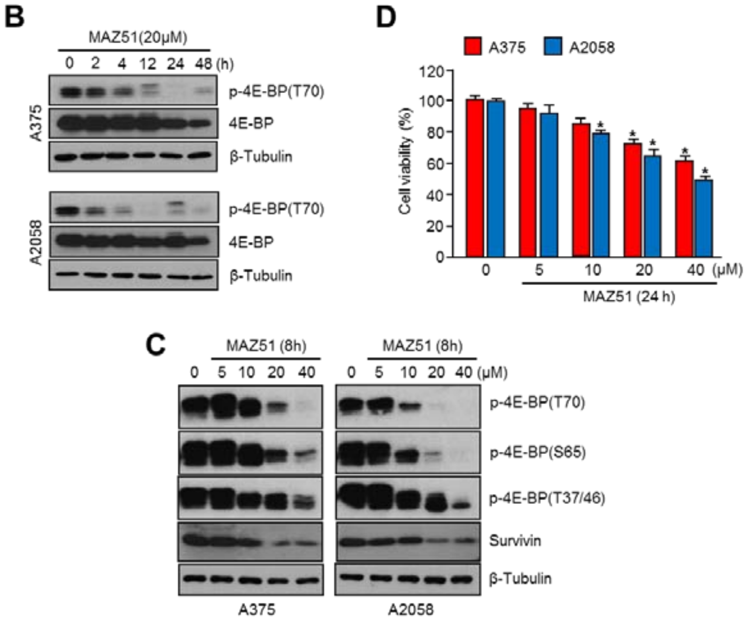

Figure S4. VEGFR3, VEGFR2, and TRKA are potential target of Fascaplysin. (**A**)The inhibitory activities of fascaplysin with respect to TRKA and VEGFR2 were measured by means of radiometric kinase assays ([γ-33P]ATP) (Reaction Biology Corp. Malvern, PA, USA); (**B**,**C**) Selective VEGFR3 inhibitor, MAZ51, suppresses 4EBP1 phosphorylation and survivin expression. Cells were incubated with 20 μM of MAZ51, and then protein levels were measured using western blotting; (**D**) MAZ51 decreases cell viability in A375 and A2058 melanoma

cells. Cells were cultured in the absence or presence of MAZ51 for 24 h, and then cell viability was measured by crystal violet staining. Values represent mean ± SD of three independent experiments performed in triplicate; # $p < 0.05$.

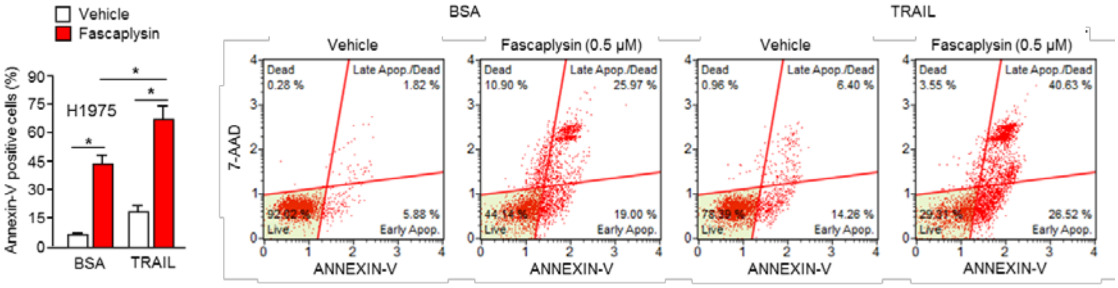

Figure S5. Fascaplysin sensitizes the anti-cancer effect of TRAIL. H1975 lung cancer cells were incubated with a combination of 0.5 μM of fascaplysin and 20 ng/ml of TRAIL for 24 h. After annexin-V staining, the population of cells was determined by FACS analysis. Values represent mean ± SD of three independent experiments performed in triplicate; * $p < 0.05$.
